# GNNSeq: A Sequence-Based Graph Neural Network for Predicting Protein–Ligand Binding Affinity

**DOI:** 10.3390/ph18030329

**Published:** 2025-02-26

**Authors:** Somanath Dandibhotla, Madhav Samudrala, Arjun Kaneriya, Sivanesan Dakshanamurthy

**Affiliations:** 1 Department of Computer Science, College of Engineering and Computing, George Mason University, Fairfax, VA 22030, USA; 2 Department of Statistics, College of Arts and Sciences, The University of Virginia, Charlottesville, VA 22903, USA; 3 Department of Computer Science, School of Computing, Data Sciences & Physics, College of William and Mary, Williamsburg, VA 23185, USA; 4 Department of Oncology, Lombardi Comprehensive Cancer Center, Georgetown University Medical Center, Washington, DC 20007, USA

**Keywords:** protein–ligand binding affinity, machine learning, graph neural network, sequence-based protein–ligand affinity prediction

## Abstract

**Background/Objectives:** Accurately predicting protein–ligand binding affinity is essential in drug discovery for identifying effective compounds. While existing sequence-based machine learning models for binding affinity prediction have shown potential, they lack accuracy and robustness in pattern recognition, which limits their generalizability across diverse and novel binding complexes. To overcome these limitations, we developed GNNSeq, a novel hybrid machine learning model that integrates a Graph Neural Network (GNN) with Random Forest (RF) and XGBoost. **Methods:** GNNSeq predicts ligand binding affinity by extracting molecular characteristics and sequence patterns from protein and ligand sequences. The fully optimized GNNSeq model was trained and tested on subsets of the PDBbind dataset. The novelty of GNNSeq lies in its exclusive reliance on sequence features, a hybrid GNN framework, and an optimized kernel-based context-switching design. By relying exclusively on sequence features, GNNSeq eliminates the need for pre-docked complexes or high-quality structural data, allowing for accurate binding affinity predictions even when interaction-based or structural information is unavailable. The integration of GNN, XGBoost, and RF improves GNNSeq performance by hierarchical sequence learning, handling complex feature interactions, reducing variance, and forming a robust ensemble that improves predictions and mitigates overfitting. The GNNSeq unique kernel-based context switching scheme optimizes model efficiency and runtime, dynamically adjusts feature weighting between sequence and basic structural information, and improves predictive accuracy and model generalization. **Results:** In benchmarking, GNNSeq performed comparably to several existing sequence-based models and achieved a Pearson correlation coefficient (PCC) of 0.784 on the PDBbind v.2020 refined set and 0.84 on the PDBbind v.2016 core set. During external validation with the DUDE-Z v.2023.06.20 dataset, GNNSeq attained an average area under the curve (AUC) of 0.74, demonstrating its ability to distinguish active ligands from decoys across diverse ligand–receptor pairs. To further evaluate its performance, we combined GNNSeq with two additional specialized models that integrate structural and protein–ligand interaction features. When tested on a curated set of well-characterized drug–target complexes, the hybrid models achieved an average PCC of 0.89, with the top-performing model reaching a PCC of 0.97. GNNSeq was designed with a strong emphasis on computational efficiency, training on 5000+ complexes in 1 h and 32 min, with real-time affinity predictions for test complexes. **Conclusions:** GNNSeq provides an efficient and scalable approach for binding affinity prediction, offering improved accuracy and generalizability while enabling large-scale virtual screening and cost-effective hit identification. GNNSeq is publicly available in a server-based graphical user interface (GUI) format.

## 1. Introduction

Binding affinity predictions between a ligand and its target protein play a crucial role in drug discovery, aiding in the identification of potential drug candidates for experimental validation. These predictions provide insight into molecular interactions and contribute to the design of more effective and selective therapeutic agents [1]. However, traditional drug discovery methods are time-consuming and costly, requiring extensive experimental testing of thousands of compounds [2]. Advances in computational methods have enabled silico screening of large chemical datasets, significantly accelerating drug discovery efforts [3]. The continuous refinement of these computational approaches has led to predictive models that are increasingly accurate and generalizable across diverse biological targets [3,4]. In particular, machine learning has emerged as a powerful tool for binding affinity prediction, offering data-driven alternatives to traditional physics-based molecular docking methods [5]. Traditional techniques often fail to capture subtle molecular properties that influence binding affinity, limiting their predictive accuracy [2,5,6]. Machine learning models overcome this limitation by efficiently parsing and analyzing protein–ligand complexes, identifying patterns that traditional techniques may overlook. Various machine learning frameworks, including Support Vector Machines (SVMs), Random Forest (RF), Convolutional Neural Networks (CNNs), and gradient boosting algorithms, have demonstrated high predictive accuracy by learning complex patterns across diverse datasets [6]. For example, CNNs and Recurrent Neural Networks (RNNs) are well suited for processing structural and sequential data, respectively, capturing intricate molecular patterns underlying binding affinity predictions [6,7,8,9]. Alternatively, Graph Neural Networks (GNNs) model protein–ligand complexes as interconnected networks, allowing them to learn multi-level feature representations that enhance predictive performance [10]. Machine learning models are also highly flexible, capable of using diverse datasets for training and predictions [11]. However, challenges such as model interpretability and the need for large, high-quality datasets persist. These limitations can be addressed through hybrid modeling approaches that combine multiple computational techniques. Combining machine learning techniques with other computational approaches has resulted in improved predictive power and reliability [12].

Amongst the different machine learning binding affinity prediction approaches, sequence-based prediction methods have emerged as a distinctive approach. Sequence-based models predict binding affinities by analyzing patterns in protein and ligand sequences, eliminating the need for detailed structural or interaction-based data. This method is advantageous because protein and ligand sequences are widely available, bypassing the requirement for molecular docking [8]. Sequence-based models, such as WGNN-DTA by Jiang et al., DeepDTA by Öztürk et al., and HNN-Denovo by Limbu et al., include the extraction of features derived from sequences, such as amino acid composition, physicochemical properties, and other sequence patterns [8,9,13]. The accessibility of these features stems from the inherent simplicity of sequence data [14,15]. However, there are still major limitations to this approach. A primary drawback is their inability to account for complex 3D protein–ligand interactions, which excludes important structural information during learning. Many of the current sequence-based models have been trained on relatively small datasets, as larger and more diverse sets are difficult to learn from [8,16]. Without proper model hybridization and optimization, limited generalizability and model overfitting may arise when a sequence-based model is applied to an unseen dataset [14]. Overcoming these limitations requires a variety of optimization techniques and algorithmic ensemble methods. In addition, integrating sequence-based models with structural or interaction-based models could improve predictive performance [9,17].

Here, we present GNNSeq, a novel hybrid machine learning method that integrates graph-based molecular representations with sequence-based features. GNNSeq leverages comprehensive feature sets for proteins and ligands, including graph features, chemical descriptors, and advanced sequence-based features, to improve binding affinity prediction. Unlike other predictive models that primarily rely on structural docking or interaction-based data, GNNSeq is designed to predict affinity exclusively from sequence features. This eliminates the need for pre-docked complexes or high-quality structural data, making it particularly useful for scenarios where structural and interaction information is unavailable or unreliable. Another predictive model, GraphscoreDTA, incorporates graph-based deep learning with a bi-transport mechanism and Vina-derived distance features, but it requires structural data for optimization, making it less applicable than GNNSeq in cases where structural information is unavailable [18]. GNNSeq integrates a Graph Neural Network (GNN) with a Random Forest (RF) Regressor and XGBoost to predict protein–ligand binding affinity. The unique combination of GNN, RF, and XGBoost in GNNSeq plays an important role in its improved predictive accuracy. The GNN component enables hierarchical sequence feature learning, while XGBoost efficiently captures complex feature interactions through gradient boosting. RF contributes to reducing variance, preventing overfitting, and enhancing model robustness. This complementary architecture allows GNNSeq to extract molecular features by incorporating both sequence-based patterns and topological properties while maintaining high generalizability across diverse protein–ligand complexes.

Trained on a diverse dataset of protein–ligand pairs from the PDBbind database, GNNSeq demonstrates strong performance and generalizability across various proteins and ligands. The model’s novel kernel-based context-switching design optimizes efficiency and runtime while dynamically adjusting feature weighting between sequence and basic structural information, making sure that the most relevant binding patterns influence the final prediction. This approach improves the model’s interpretability. GNNSeq was developed with a strong emphasis on computational efficiency and enables large-scale predictions without compromising runtime. Other sequence-based predictive models have demonstrated strong performance but differ fundamentally from GNNSeq in their approach. ISLAND is an SVM-based sequence-only predictor that relies on handcrafted features like amino acid composition and evolutionary profiles, while GNNSeq leverages graph-based learning and ensemble modeling for improved generalization [19]. DeepDTA, a CNN-based model, extracts protein–ligand features from sequence data but lacks the hierarchical feature integration and ensemble learning framework that GNNSeq employs to enhance feature interactions and generalization [20]. Additionally, integrating GNNSeq with structural- and interaction-based models improved its predictive power for both known and de novo complexes. GNNSeq overcomes key limitations of traditional sequence-based models, such as issues with accuracy, robustness, generalizability, efficiency, and adaptability, for integration with other frameworks.

## 2. Results and Discussion

**Figure 1:**
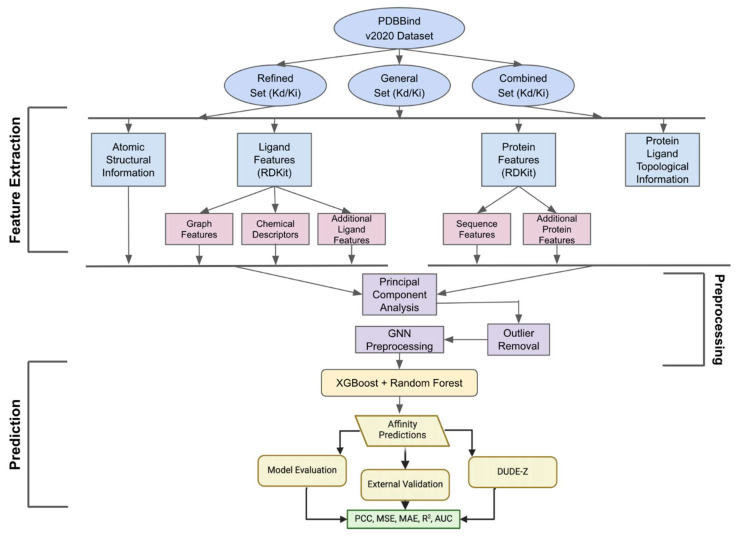
Overview of GNNSeq workflow. The GNNSeq workflow begins with the PDBbind v.2020 dataset, categorized into refined, general, and combined sets. Feature extraction derives atomic structural information, ligand features such as graph features and chemical descriptors, protein features such as sequence features, and combined protein–ligand topological information. After feature extraction, PCA is applied to reduce dimensionality, and outlier removal is performed. The features are then processed through the Graph Neural Network (GNN), XGBoost, and Random Forest for binding affinity predictions. The workflow concludes with model evaluation of GNNSeq, followed by external validation using the DUDE-Z v.2023.06.20 dataset. The model is assessed using the performance metrics PCC, MSE, MAE, R^2^, and AUC.

This study presents GNNSeq, a robust sequence-based predictive model that combines feature extraction, hybrid modeling, and comprehensive validation to accurately predict protein–ligand binding affinities. GNNSeq integrates a Graph Neural Network (GNN) with Random Forest (RF) and XGBoost to capture complex sequence patterns within protein–ligand complexes. The workflow begins with the PDBbind v.2020 dataset, organized into refined, general, and combined subsets (Figure 1). Features are extracted to represent atomic-level structural details, ligand properties (e.g., graph features, chemical descriptors), protein sequence data, and combined protein–ligand topological information. Graph-based features, such as node degrees, clustering coefficients, and betweenness centrality, provide detailed structural representations of ligands, enabling the model to identify subtle variations impacting binding interactions [8,16]. Protein sequence-based features, including amino acid frequencies, hydrophobicity, polarity, and secondary structure fractions, capture the biochemical environment of binding pockets [21]. Atomic and molecular-level structural features, extracted using RDKit v. 2024.03.4, are integrated into the graph representation to model protein–ligand conformations. After feature extraction, dimensionality reduction (PCA) and outlier removal optimize the data. The processed features are input into the GNN and combined with RF and XGBoost to predict binding affinities. GNNSeq performance is evaluated using metrics like PCC, MSE, MAE, R^2^, and AUC, followed by external validation with the DUDE-Z v.2023.06.20 dataset to assess robustness and generalizability.

### 2.1. K-Fold Cross-Validation Results on the Refined Set

We began our first evaluation of GNNSeq’s performance with training and testing on the refined set. GNNSeq achieved the following performance metrics across ten folds. As indicated by the average R^2^ score, MSE, MAE, PCC, and AUC, GNNSeq consistently performed well across different randomized subsets of the “refined” set. The R^2^ score averaged 0.595 across the folds. The MSE and MAE were 1.524 kcal/mol and 0.963 kcal/mol, respectively. AUC is conventionally used as a classification metric and is derived from a receiver operating characteristic (ROC) curve based on true positive rate (TPR) and false positive rate (FPR). However, since PDBbind is a regression dataset, a direct classification-based AUC calculation is not applicable. Instead, AUC was computed using a median-based thresholding approach to approximate classification performance within a continuous affinity dataset.

**Figure 2:**
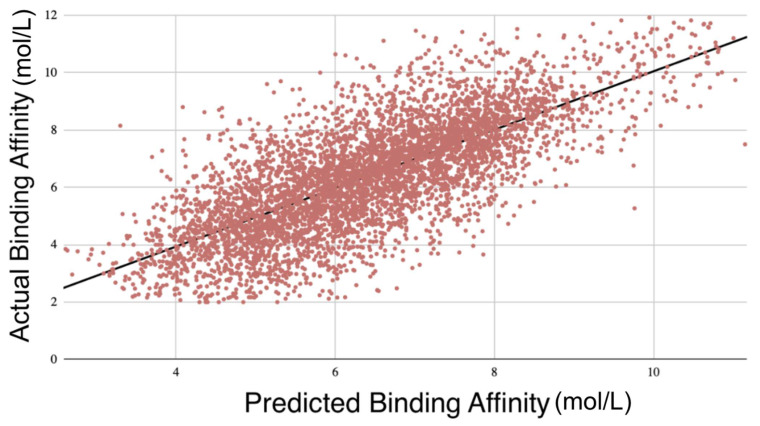
Scatterplot showing an example run of predicted vs. actual binding affinities, generated using k-fold cross-validation on the PDBbind refined set with z-score outlier removal.

To achieve this, binding affinity values were split into two groups: complexes with binding affinity above the median were labeled as high-affinity, while those at or below the median were labeled as low-affinity. The model’s predicted affinity values were thresholded in the same way, and AUC was then computed to evaluate how well the model distinguished between these two groups. This allows for a meaningful interpretation of AUC in a regression context by assessing whether the model correctly separates stronger from weaker binders. The AUC averaged 0.792 and the PCC averaged 0.784, demonstrating a strong correlation (Table 1, Tables S1 and S2). Cross-validation was performed similarly five times to confirm the reproducibility of GNNSeq’s predictions and accuracy (Figure 2). The entire 10-fold cross-validation took an average of 85 min. This efficiency is due to the hyperparameter optimization, as well as the ensemble approach of the GNN, RF, and XGBoost.

### 2.2. K-Fold Cross-Validation Results on the General Set

After the refined set, we moved to a larger, more diverse general set. GNNSeq achieved the following performance metrics across ten folds. Based on the average R^2^ score, MSE, MAE, PCC, and AUC, GNNSeq demonstrates consistent performance across the ten folds in the “general” set. The R^2^ score averaged 0.479, suggesting moderate predictive power. The MSE and MAE were 1.885 kcal/mol and 1.068 kcal/mol, respectively. The average PCC of 0.718 indicates a reasonably strong linear correlation between predicted and actual affinities, while the AUC, at 0.760, shows the robustness of GNNSeq (Table 2 and Table S3). These results indicate a slight drop in performance on the general set compared to the refined set due to the increased diversity of complexes [9]. The increased diversity introduces greater variability, making it harder for the model to identify consistent patterns and generalize accurately across all complexes. The cross-validation process was repeated five times to confirm the consistency and accuracy of GNNSeq’s predictions. The full 10-fold cross-validation was completed in an average of 241 min due to hyperparameter optimization and the efficient ensemble of the GNN, RF, and XGBoost models.

### 2.3. K-Fold Cross-Validation Results on the Combined Set

Following the general set, we merged the refined and general sets to create a more holistic combined set. GNNSeq consistently demonstrates strong performance across tenfolds of training and testing with the combined set. The average R^2^ score was 0.509, indicating a strong correlation. The MSE and MAE averaged 1.778 kcal/mol and 1.039 kcal/mol, respectively, reflecting accuracy in predictions. The PCC averaged 0.740, showing a robust correlation between predicted and actual binding affinities. Finally, the AUC averaged 0.776, indicating reliable classification performance (Table 3, Table S4). The combined set provides an even more diverse base than the “general” set due to the fact that there is a greater number of complexes with a broad range of binding affinities. This makes it suitable to be the main training set for GNNSeq, as the model can learn and analyze patterns from more complexes [8]. The complete 10-fold cross-validation took an average of 114 min.

### 2.4. Using the Refined and General Sets for External Validation of GNNSeq

**Figure 3:**
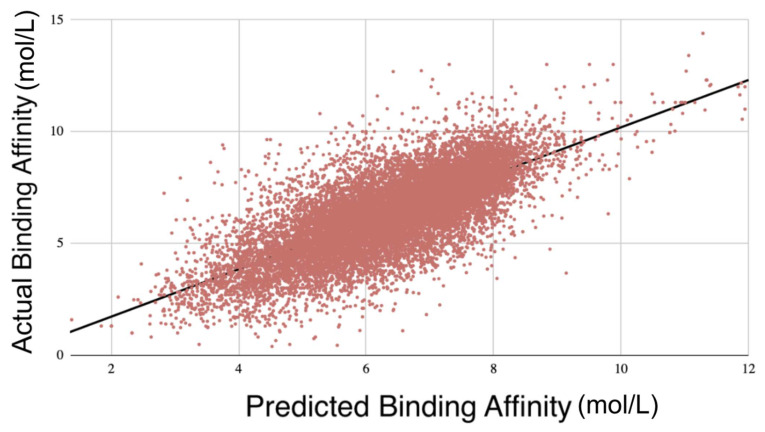
Scatterplot of an example external validation run. The model was trained on the “PDBbind” refined set and externally validated using the PDBbind v.2020 general set. The graph shows the predicted binding affinities vs. the actual binding affinities using K-fold cross-validation while training on the PDBbind refined set and z-score outlier removal.

After training the model using 10-fold cross-validation, we performed external validation using the refined and general sets to gauge GNNSeq’s ability to make predictions based on unseen datasets. GNNSeq_1_ was trained on the refined set and validated on the general set, while GNNSeq_2_ was trained on the general set and validated on the refined set. We ran these tests three times for reproducibility with the average metrics over these three runs. Despite the increased complexity and variability in the general set, the model maintained a reasonable level of predictive accuracy. GNNSeq_1_ showed lower metrics with a PCC of 0.612 and R^2^ of 0.373. This is expected, as the general set has a broader diversity of protein–ligand complexes, making it harder for a model trained on the less diverse refined set [9]. GNNSeq_2_, trained on the general set and validated on the refined set, had higher metrics, with a PCC of 0.687 and R^2^ of 0.461 (Table 4, Figure 3, Table S5). This shows that training on the more diverse general set allows the model to learn about a wider range of complexes, which improves its ability to generalize. These results also prove the model’s robustness and applicability to diverse datasets.

### 2.5. External Validation Using the DUDE-Z Dataset

Next, we performed external validation using the DUDE-Z v.2023.06.20 dataset. The DUDE-Z dataset, a successor to the DUD-E database, features 43 receptors with native crystal structure ligands and thousands of active and decoy ligands. The DUDE-Z v.2023.06.20 dataset was used to rigorously validate the predictive capabilities of GNNSeq, with a specific focus on its ability to accurately distinguish between active and decoy ligands. The active ligands were labeled as “CHEMBL” and the decoy ligands were labeled as “ZINC”. To show the distinction between the active and decoy ligands, the mean predicted values for CHEMBL and ZINC ligands were calculated for each receptor. The differences in these values provide insight into GNNseq’s discriminatory power.

**Figure 4:**
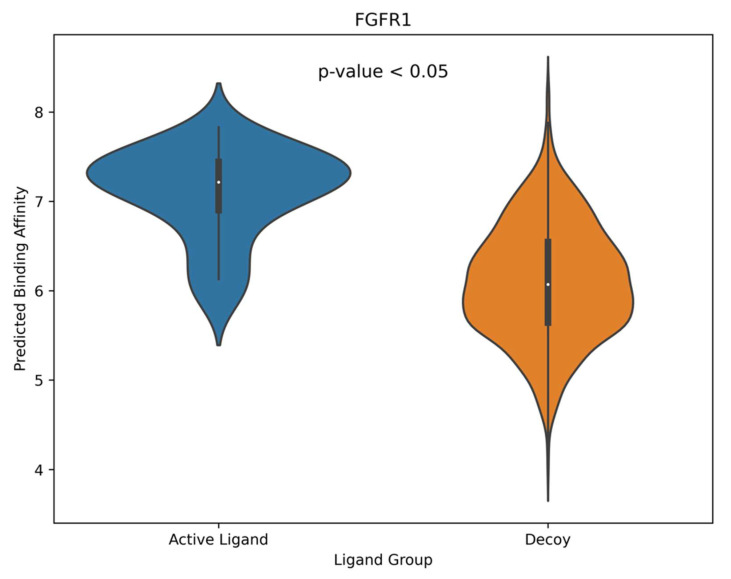
A sample violin plot comparing the predicted binding affinities of active ligands and decoys for the FGFR1 receptor. The active ligands exhibit higher and more consistent predicted binding affinities, while the decoys show a broader range with lower median values. The *p*-value < 0.05 shows that the difference between the two groups is statistically significant.

GNNSeq outputs binding affinity predictions as −log (K_d_/K_i_) values, which lists all receptor–ligand pairs with predicted affinities in −log (Kd/Ki) and micromolar (µM) units. To evaluate GNNSeq’s ability to distinguish active from decoy ligands, we visualized the predictions using violin plots and performed t-tests between active and decoy ligand predictions. Violin plots illustrate binding affinity predictions for active and decoy ligands, with wider sections highlighting areas of higher prediction density. The *p*-value displayed in the top results from t-tests compares mean predictions between active and decoy ligands. Binding affinities were plotted on the *y*-axis with a threshold of 6 (−log (K_d_/K_i_)) or 1 µM to classify true actives versus decoys. Values of 6 and above are considered active, while those below indicate decoys. A sample violin plot of the FGFR1 receptor can be seen in Figure 4. The violin plot shows that active ligands have higher and more consistent predicted binding affinities compared to decoys, as indicated by the tighter distribution and higher median in the active ligand group. A *p*-value < 0.05 indicates a statistically significant difference between active decoy ligand predictions by GNNSeq.

Looking at the violin plots, we can see that GNNSeq predicts that active ligands bind stronger to their receptors than the decoy ligands across all 43 receptors in the whole DUDE-Z v.2023.06.20 dataset (Table S6). These results are statistically significant, as indicated by a t-test *p*-value below 0.05. A positive difference between the CHEMBL mean and ZINC mean indicates successful differentiation, with higher positive values correlating with greater model confidence in identifying true binders. The model consistently produced positive differences, reinforcing its robustness and reliability in a diverse array of protein–ligand interactions. This indicated that the model effectively differentiated between true binders and decoys [22]. This is important because the DUDE-Z v.2023.06.20 dataset presents a significant challenge, as decoys are specifically chosen to closely resemble active ligands in structure. This design tests the model’s ability to identify subtle yet crucial differences between active and decoy ligands. The strong predictive performance observed in this study implies that the model is not only capable of handling the complexity of large-scale datasets but that it can also provide reliable predictions.

### 2.6. Benchmarking GNNSeq Against Similar Deep Learning Methods

Next, we carried out a comparative analysis to benchmark the predictive performance of GNNSeq against various related deep-learning methods reported in the literature. The comparison uses PCC, MSE, and MAE as evaluation metrics. The primary objective of this comparison is to understand how effective GNNSeq’s predictive power is against existing methods used to predict protein–ligand binding affinity. The comparison includes a range of machine learning and deep learning methods, such as Random Forest-based models, various GNN approaches, and other related or novel neural network architectures. Each method reported has been evaluated using different versions of the PDBbind dataset (Table 5). Performance metrics of external tools were taken from the original publications. The metrics from the studies being compared show their model performance on external test datasets, which provide a more holistic view of the model’s ability to generalize. We used the PDBbind v.2019 refined set, the PDBbind v.2016 refined set, the PDBbind v.2016 core set, and the PDBbind v.2013 core set to compare the models. The core sets are slightly undiversified sets of about 200 complexes that contain 3D crystal structures with extremely high resolution.

GNNSeq achieved competitive performance in terms of PCC, MSE, and MAE on various PDBbind refined and core sets. In the PDBbind v.2020 refined set, GNNSeq achieved a PCC of 0.784, an MSE of 1.51 kcal/mol, and an MAE of 0.957 kcal/mol. On the v.2019 refined set, it achieved a PCC of 0.771, with an MSE of 1.56 and MAE of 0.988. In comparison, GNNSeq’s performance on the PDBbind v.2016 core set showed a higher PCC of 0.839, with a slightly higher MSE of 1.665 kcal/mol and MAE of 1.001 kcal/mol (Table 5). When compared to current machine learning models evaluated on the PDBbind v.2016 core set, GNNSeq remains highly competitive. Models such as HAC-Net (PCC = 0.846, MAE = 0.971 kcal/mol) [25], AEScore (PCC = 0.83) [27], and EHIGN (PCC = 0.854) [31] achieve similar performance levels. Notably, GraphBAR (PCC = 0.726, MAE = 1.241 kcal/mol) [30] and BAPA (PCC = 0.819, MAE = 1.021 kcal/mol) [29] perform worse than GNNSeq in terms of PCC, likely due to their reliance on feature representations that may not generalize well across diverse protein–ligand interactions. In contrast, T-ALPHA (PCC = 0.869, MAE = 0.875 kcal/mol) [32] and TopoFormer-Seq (PCC = 0.864) [33] marginally outperform GNNSeq, potentially due to their use of transformer-based architectures that capture long-range dependencies more effectively. Despite this, GNNSeq achieves comparable performance while relying solely on sequence-based features, demonstrating its efficiency and robustness in binding affinity prediction. In comparison to other more diverse sets, while models like HNNAffinity [9] achieved a higher PCC of 0.830 on the PDBbind v.2019 refined set, and MP-GNN [24] reached 0.805 on the PDBbind v.2013 core set, GNNSeq remains competitive with PCC values close to these models (Table 5). GNNSeq demonstrates that it is highly competitive even when compared with other models such as SS-GNN [24], which scored a PCC of 0.795 on the PDBbind refined set. This is achieved despite primarily relying solely on sequence-based features. This performance indicates that GNNSeq can efficiently provide meaningful predictions using only sequence data and compete with far more complex models.

### 2.7. Performance of GNNSeq Algorithmically Hybridized with CNN, RF, and XGBoost

After we benchmarked GNNSeq against existing models, we tested various algorithmic hybridizations of GNNSeq on the PDBbind v.2020 refined set. These algorithmic hybridizations were with CNN, RF, and XGBoost. These different hybridized models were developed and tested individually. To ensure that each hybrid model operated at its peak performance, Hyperopt v.0.2.7 was used to fine-tune the hyperparameters for all hybrids [38]. The results presented are accurate and reproducible. Each model was trained and tested three times, using 10-fold cross-validation on the PDBbind v.2020 refined set. The primary goal was to identify the hybrid sequence-based model that offers the highest accuracy and computational efficiency, making it the optimal choice for the final version of GNNSeq.

The results from this test show the performance of various algorithmic hybrids for GNNSeq’s sequence-based protein–ligand binding affinity prediction on the refined set. The CNN + GNN hybrid achieved a PCC of 0.72, an AUC of 0.74, and a runtime of 6 h and 6 min. The standalone CNN performed slightly worse, with a PCC of 0.66, an AUC of 0.72, and a runtime of 3 h and 37 min. The GNN + CNN + XGBoost hybrid reached a PCC of 0.744 and an AUC of 0.76, with a longer runtime of 6 h and 47 min. GNN + CNN + RF recorded a PCC of 0.741, an AUC of 0.73, and a runtime of 6 h and 41 min. The CNN + XGBoost + RF hybridization had a PCC of 0.687, an AUC of 0.70, and a runtime of 6 h and 8 min. Finally, the GNN + CNN + XGBoost + RF hybrid achieved a PCC of 0.783, an AUC of 0.749, and a runtime of 8 h and 49 min (Table 6).

Among the tested combinations, the GNN + XGBoost + RF hybrid emerged as the top performer, achieving a PCC of 0.784 and an AUC of 0.792, with an efficient runtime of 1 h and 32 min. This ensemble provided the highest accuracy and the lowest runtime amongst all hybrids, making it the optimal method. This also demonstrates that it can be feasibly integrated into larger workflows and frameworks without excessive computational overhead. The integration of GNN, XGBoost, and Random Forest in this study uses the strengths of each method to create a robust and accurate predictive model. The efficiency and accuracy of this hybridization are due to the GNN’s ability to take into account complex sequence-based patterns and the efficiency of RF and XGBoost in handling diverse datasets. Using a GNN allows the usage of the structural information inherent in protein and ligand sequences. By converting these sequences into graph representations, the GNN allows the model to learn complex relationships and dependencies that may be missed during regular feature extraction. Random Forest is effective in handling large, highly dimensional datasets with many features. Using RF provides robust predictions as it reduces variance and prevents overfitting. XGBoost, another ensemble technique based on gradient boosting, offers strength to the model by optimizing the use of bias and variance. It provides high performance in predictive accuracy due to its ability to handle a wide range of loss functions and provides efficient computation using parallel processing. The combination of RF, XGBoost, and the GNN creates an ensemble, where the strengths of each method complement the others. This results in a model that is both accurate and generalizable. RF and XGBoost contribute to the model’s robustness and predictive power, while the GNN makes sure that the model maps the essential features of the necessary sequences into a traversable graph. The selected combination of RF, XGBoost, and GNN represents a strategic choice aimed at maximizing the model’s efficiency and accuracy in predicting binding affinities.

The GNN + XGBoost + RF hybrid model was chosen over alternative hybridized frameworks like CNNs. Although CNNs excel at detecting spatial hierarchies in data, they rely heavily on large amounts of labeled data. The standalone predictive CNN we developed was initially integrated into the hybrid models for its ability to capture spatial hierarchies and local patterns within the protein–ligand complexes. However, CNN-based hybrids did not perform as well as anticipated and slowed computational time significantly. The GNN + CNN + XGBoost + RF combination yielded a PCC of 0.783 and an AUC of 0.749. This is slightly lower than the GNN + XGBoost + RF hybrid, which means that the inclusion of the optimized CNN added complexity without a corresponding increase in predictive power. The addition of the CNN caused the model to struggle with the specific feature sets used in this study, resulting in a decline in prediction accuracy. The runtime for this model was 8 h and 49 min due to the significant workload from the CNN layers. A similar trend was observed with other CNN hybrids (Table 6). These models were each run multiple times to confirm that the addition of CNNs caused a significant increase in computation time. This shows that while CNNs may contribute to increasing the model’s robustness, they do not improve overall predictive accuracy. The PCC scores were consistently lower by about 0.08 to 0.10 compared to non-CNN hybrids, and the substantial increase in runtime detracts from their practicality in this study.

### 2.8. Assessing the Integration of an Interaction-Based Model

Next, we attempted to merge GNNSeq with an interaction-based model in an effort to improve predictive capabilities. Interaction-based models excel in capturing detailed protein–ligand interactions, which are critical for understanding the binding mechanisms at a low level. However, these models require comprehensive structural or interaction data, which may not always be available. This limits their standalone capabilities. Sequence-based models can predict binding affinity directly from amino acid sequences, making them more versatile. Yet, their downside is the lack of precision that detailed interaction data and features offer, which makes a combined approach an ideal solution to leverage the strengths of both. The interaction-based model, labeled Int_alg_, primarily uses chemical protein–ligand interaction data to make binding affinity predictions.

As an average of three runs, the standalone GNNSeq model yielded a PCC of 0.784 and an AUC of 0.792. These metrics serve as a control to assess the effectiveness of the integration techniques. In the first merging strategy (V1), where the interaction-based model was hybridized with the sequence-based model using basic concatenation of embeddings, the performance slightly decreased with a PCC of 0.788 and an AUC of 0.780 (Table 7). This drop may be attributed to the failure of the hybridized embeddings to effectively eliminate redundancies in model features. This reduction in PCC is intuitive, as simply concatenating the embeddings of two separate approaches does not holistically allow the merged model to learn effectively.

After the first baseline merge was performed, a more sophisticated approach (V2) involving a four-layer stacking of interaction predictions was used. This approach led to a slight improvement in PCC (0.792), with an AUC of 0.790. The improved handling of interaction data via stacking likely allowed the model to better generalize the complementary information from both models. Building on the improvements that came from stacking, the most significant boost in performance came with the introduction of a kernel-level optimizer in V3, which algorithmically optimized context switching and maintained runtime efficiency. This kernel-level strategy resulted in an improved PCC of 0.802 and an AUC of 0.790. Finally, the fully optimized kernel-level stacking method (V4), which integrated interaction predictions and stacked embeddings, achieved the highest performance, with a PCC of 0.826 and an AUC of 0.805 (Table 7). This shows that more complex and refined merging strategies, when coupled with kernel-level programming, can significantly improve both accuracy and robustness without leading to exponential increases in runtime.

In V1, the straightforward concatenation of embeddings did not yield significant improvements due to potential redundancies or conflicts between the feature sets. In stacking (V2) and kernel-level optimization (V3), the merging process was refined to allow for the strengths of models to complement each other. Kernel-level programming further improved merging by guaranteeing efficient context switching. Context switching allowed for the integration of deeper layers without a significant increase in runtime. V4 achieved the highest accuracy by integrating interaction predictions and embeddings with Hyperopt v.0.2.7 tuning and kernel-level programming [38]. This reinforces that integrating interaction data with sequence data can expand the model’s learning capacity and improve prediction accuracy. However, the hybrid model’s predictive effectiveness largely depends on the quality and efficiency of the merging techniques used.

### 2.9. Assessing the Integration of a Structure-Based Model

Here, we merged GNNSeq with an external structure-based predictive GNN model named Struct_alg_. The structure-based model, Struct_alg_, primarily uses structural features and topological data to make binding affinity predictions. Structure-based models, in general, focus on capturing 3D spatial relationships and geometric features of the protein and ligand. However, structural data alone can sometimes fail to generalize across different protein–ligand pairs, especially when the structures are either unknown or difficult to obtain. In contrast, sequence-based models are not limited by the availability of 3D structures and can operate on basic sequence data. While they are less precise, this flexibility allows sequence-based models to have broader applicability. Due to the strengths of both approaches, a combined method leverages the precision of structural features from Struct_alg_ and the versatility of sequence data from GNNSeq when both sequence and structure data are available.

Averaged over three runs, the PCC and AUC for the standalone GNNSeq model remained at 0.790 and 0.791, respectively. However, when the structure-based model was hybridized with the sequence model (V1), it yielded a PCC of 0.721 and an AUC of 0.741 (Table 8). This decline shows that structural features, when not merged well, may decrease the predictive power of sequence-derived features. This is particularly true if the structural data are not fully complementary to the sequence-based information [8,10]. This mismatch likely resulted in feature-based learning conflicts that reflected a decreased predictive performance. Improvements were observed with the V2 strategy, which introduced a three-layer stacking technique along with an additional hidden layer. This approach increased the PCC to 0.748 and the AUC to 0.770, which demonstrated that the added hidden layer improved the integration of structure-based features. The third strategy (V3) applied a similar kernel-level optimizer to the hybridized structure-based GNN, which resulted in a PCC of 0.759 and an AUC of 0.790. This performance boost can be attributed to kernel-level optimization. Kernel-level optimization ensures that context switching between the sequence and structure models is efficient and avoids significant runtime increases. Finally, kernel-level stacking of structure predictions and embeddings with Hyperopt v.0.2.7 tuning (V4) achieved the best performance, with a PCC of 0.795 and an AUC of 0.802 (Table 8). This confirms that sophisticated stacking methods, when combined with optimizations at the kernel level, lead to better predictive performance of the structure-based model. V4 performs similarly to the standalone GNNSeq model, suggesting that the structure-based model primarily contributed additional structural information to the sequence-based model. However, the accuracy growth trend for the structure-based model among merge versions shows that with more targeted merging techniques, these models may perform better.

### 2.10. Algorithmically Merging Sequence, Structure, and Interaction-Based Models

Once we had merged GNNSeq with the interaction and structure-based models separately, we attempted to merge all three models together to improve predictive abilities. The rationale behind merging the sequence-based, structure-based, and interaction-based models was to create a comprehensive framework that leverages the strengths of each model type. Sequence-based models provide a baseline level of predictive power that is not dependent on detailed structural or interaction data, which makes them highly flexible. Structure-based models, as discussed earlier, add geometric precision when 3D data are available. This enriches the model’s ability to understand the spatial relationships between proteins and ligands. Interaction-based models contribute highly specific information about molecular interactions. These are essential for understanding the intricacies of binding mechanisms. By integrating these three types of models, the goal was to develop a highly robust and accurate framework capable of predicting binding affinity across a wide range of protein–ligand pairs, even when only partial information is available.

The baseline performance for the standalone GNNSeq, averaged over three runs, resulted in a PCC of 0.790 and an AUC of 0.791. The first merging attempt, (V1), hybridized the structure-based and interaction-based GNNs with the sequence model by concatenating the embeddings. This resulted in a slight decline in performance, with a PCC of 0.789 and an AUC of 0.772 (Table 9). This outcome indicates that the direct combination of these models did not effectively use the complementary strengths of each, similar to the individual merges. By adopting a more advanced stacking approach (V2), which involved seven layers and one hidden layer, the model’s performance significantly improved, achieving a PCC of 0.821 and an AUC of 0.804. This indicates that the increased complexity of the stacking technique was crucial in extracting useful features from the combined models. The introduction of a kernel-level optimizer in V3 further boosted performance to a PCC of 0.828 and an AUC of 0.815, again due to the efficiency of the context switching being nearly linear. The best-performing merge, V4, involved kernel-level stacking of structure, sequence, and interaction predictions across eight layers with Hyperopt v.0.2.7 tuning [38]. V4 obtained a PCC of 0.839 and an AUC of 0.819 after testing (Table 9).

### 2.11. Kernel-Level Optimizations and Merging Techniques

A key innovation in this study lies in the use of kernel-level programming and permissions, which play an important role in optimizing the efficiency of the merging process between the sequence-based, structure-based, and interaction-based models. Traditional machine learning models often experience exponential increases in runtime as additional layers or more complex stacking methods are added on. By shifting kernel permissions and using custom kernel-level optimization to optimally change when context switches in the OS occur, we ensure that the runtime only increases linearly, regardless of the number of layers or the complexity of the stacking technique [39].

This optimization is achieved through a reduction in the rate of context switching between different parts of the program, mitigating a common bottleneck in high-level computations involving multiple processes. By algorithmically managing these context switches, we reduced the overhead typically associated with these operations. This improvement is important when working with large-scale datasets, such as the PDBbind and DUDE-Z datasets. The use of kernel-level programming makes the merging process more scalable and guarantees that increased complexity in the model architecture does not significantly increase the runtime. Kernel-level optimization allowed the runtime to remain under 2 h, regardless of how many models we merged. The custom-developed algorithm was able to choose when to context switch based on the number of processes remaining on the stack from each model. This saves the overall merged models many fractional amounts of time, which allows the increase in runtime to stay linear for the most part. The resultant efficiency allows for more intricate merging strategies to be used without sacrificing computational feasibility. This is one of the key features that distinguishes this merging technique from others. Specific permissions in the computer running the code must be given to run or alter the kernel-level merges. Kernel context switching cannot be used for the stand-alone GNNSeq since there is no other overarching process to context switch to.

In addition to runtime improvements, the chosen merging strategy itself affects the model’s accuracy and robustness. As Table 7, Table 8 and Table 9 indicate, merging techniques involving kernel-level optimizations generally lead to improved predictive performance. This shows that the complexity of the merging technique improves the model’s ability to identify patterns in the data, and the choice of merging algorithm significantly influences the model’s overall performance. Overall, the results demonstrate that advanced merging methods can improve prediction accuracy and robustness by optimizing information flow between models.

### 2.12. Evaluating the Performance of All Hybrid Merges on Well-Known Drugs

**Figure 5:**
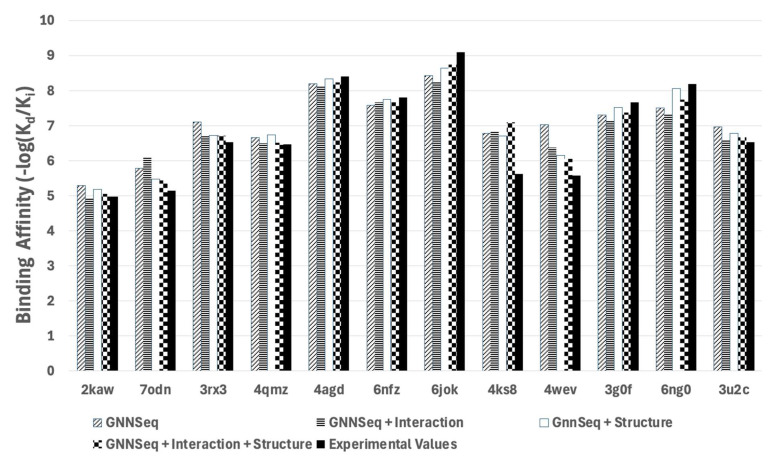
A side-by-side bar chart displaying the binding affinity predictions in −log (K_d_/K_i_) for the hybrid models compared with their experimental values.

To further validate the effectiveness of the combined models, the advanced merging strategies were tested on protein–ligand complexes with three well-known drugs: mebendazole, sulindac, and sunitinib. For this validation, we used the structures of these drugs docked with their respective protein targets from the RCSB and compared predictions with experimental binding affinity values obtained from BindingDB. The model variations tested included GNNSeq alone, GNNSeq + *Int_alg_*, GNNSeq + *Struct_alg_*, and GNNSeq + *Int_alg_* + *Struct_alg_*. This evaluation focused on twelve protein–ligand complexes representing all hybrid model combinations for each drug. The PDB codes 2kaw, 3rx3, 3u2c, and 4wev correspond to complexes containing the drug sulindac. Sunitinib is represented by the PDB codes 3g0f, 4agd, 4ks8, 4qmz, 6jok, 6nfz, and 6ng0. The PDB code 7odn is a PDB complex containing mebendazole (Figure 5, Table S7).

All the models provided highly accurate binding affinity predictions (Figure 5, Table S7). The experimental results indicated that while all models provided binding affinity predictions near the experimental values, the GNNSeq + *Int_alg_* + *Struct_alg_* model consistently achieved predictions closest to the experimental values (Figure 5, Table S7). GNNSeq alone performed comparably well, demonstrating the effectiveness of sequence-based prediction on new data even when structure data are unavailable. While GNNSeq with sequence features excelled in the training phase, the structural enrichment in GNNSeq + *Struct_alg_* improved generalization for external validation, likely due to its ability to capture spatial conformations more effectively. The GNNSeq + *Int_alg_* model performed well to already seen protein–ligand predictions but did not improve underlying learning mechanisms to the same extent for mebendazole, sulindac, and sunitinib.

### 2.13. Evaluating All Hybrid GNNSeq Predictions Against AutoDock Vina Predictions

To assess the accuracy of GNNSeq predictions, we compared the hybrid GNNSeq predictions against the binding affinity predictions from AutoDock Vina 1.1.2 and experimental binding affinities for several docked compounds. We used the Gibbs Free Energy Change equation to convert the free energy value that AutoDock Vina 1.1.2 produces during the docking process into binding affinity. These calculated binding affinities were then compared to GNNSeq’s predictions to evaluate which were closer to the experimental values.


(1) $$K_{d}=e^{\Delta G/RT}$$

**Figure 6:**
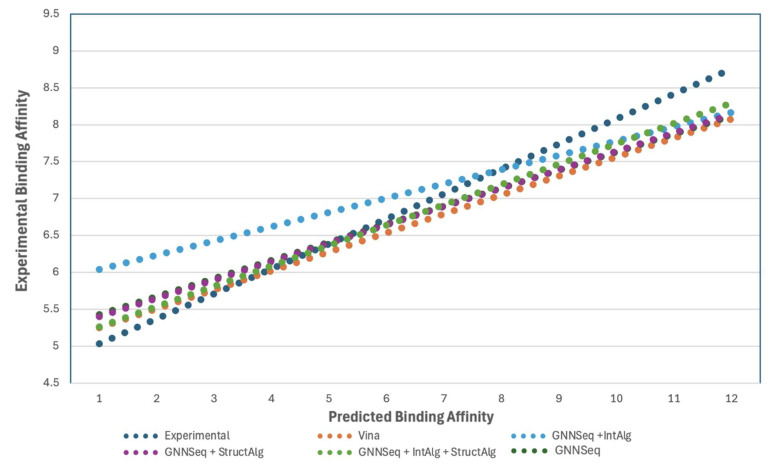
Linear trendlines comparing experimental and predicted binding affinities for different hybrid models: GNNSeq, GNNSeq + Stuct_alg_, GNNSeq + Int_alg_, GNNSeq + Int_alg_ + Struct_alg_, and AutoDock Vina 1.1.2. Each trendline is based on predictions for 12 complexes docked with Vina. Binding affinities are shown in −log (K_d_) for improved linear fits.

The standalone GNNSeq model achieved a PCC of 0.73 for the docked compounds. When hybridized with the interaction-based model, the PCC improved to 0.76. Hybridizing GNNSeq with the structure-based model resulted in a PCC of 0.74. The highest performance was achieved with the combined hybrid model, integrating GNNSeq with both interaction- and structure-based models, which attained a PCC of 0.82 (Figure 6). These results demonstrate that GNNSeq and its hybridized forms provide accurate binding affinity predictions and generally perform better than Vina’s binding affinity outputs when compared to the experimental data. The linear trendlines represent the relationship between predicted and experimental binding affinities for each model, which shows how well the predictions align with the experimental reference line. Closer alignment to the reference line indicates higher prediction accuracy. The real-time binding affinity simulations with predictions for the 12 docked complexes can be viewed in the GUI at https://github.com/sivaGU/GNNSeq.

### 2.14. Development of GNNSeq GUI

To display the results of the study in a more streamlined fashion, we created a graphical user interface (GUI) named GNNSeq GUI. Based on user input, this GUI retrieves the necessary files and predicts binding affinities in real-time. Slight variations (~±0.03) in repeated predictions for the same complex can be attributed to stochastic processes inherent in the model’s operations, such as randomized initialization or sampling during inference. Since the model treats each prediction as independent, these minor fluctuations are negligible and do not affect overall reproducibility or reliability. This variation reflects the natural variability in machine learning-based predictions.

**Figure 7:**
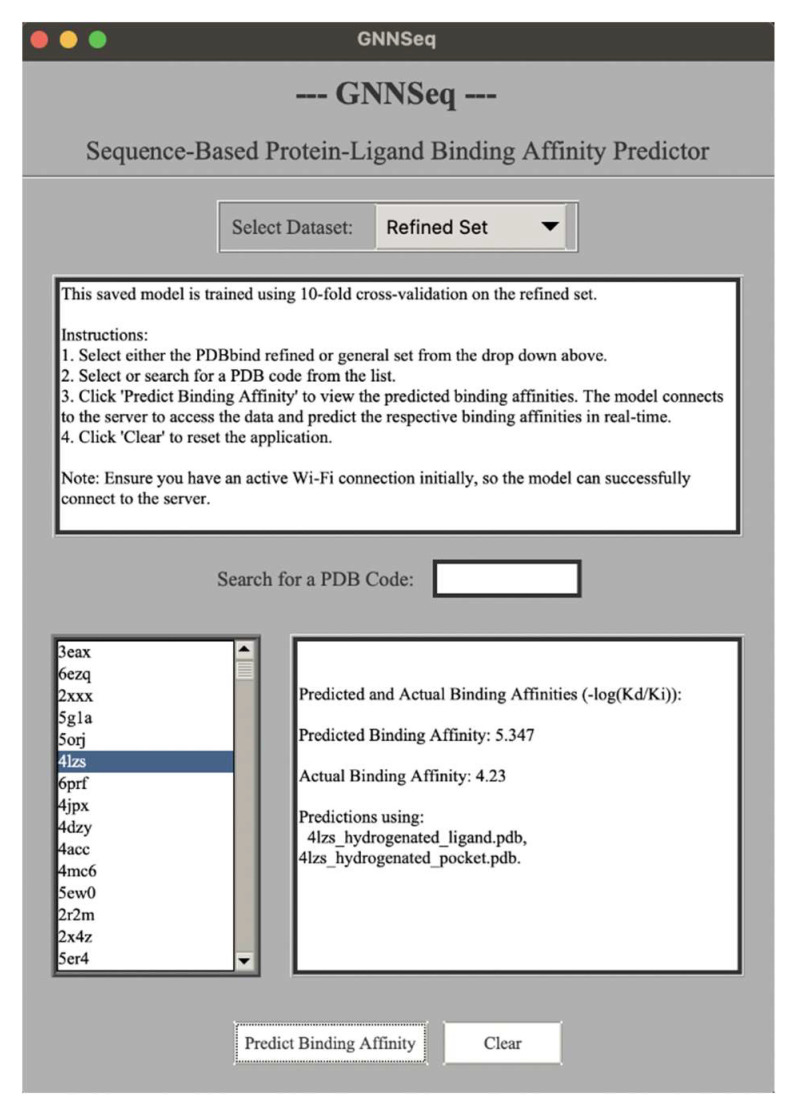
A Screenshot of the GNNSeq GUI, illustrating its real-time prediction workflow. The GUI loads trained models locally, connects to a server to access protein–ligand complex files based on user input, and outputs binding affinity predictions with slight stochastic variation.

The GNNSeq GUI provides an intuitive interface for predicting protein–ligand binding affinities (Figure 7). The interface has a dropdown menu that toggles between three datasets: the refined set, the general set, and the small set of virtually docked complexes by AutoDock Vina 1.1.2. Detailed instructions for using the GUI are provided in the display box at the top of the GUI, guiding users through the workflow. The user can begin by selecting a dataset from the dropdown menu, searching for or selecting a PDB code or docked complex name, and then running the prediction process. The display box on the left shows the list of all the available PDB codes for the selected refined or general set and the names of the docked complexes for the docked complexes dataset. Users can scroll through the list and select a specific PDB code or complex name by clicking on it. To improve usability, a search bar is provided above the list of PDB codes and complex names. This feature allows users to search for a specific PDB code or docked complex name within the selected dataset. As the user types, the list dynamically updates to show matching or partially matching entries. If there is no matching entry, the GUI provides a “No match is found” message, after which the user can refine their search.

The “Predict Binding Affinity” button will trigger the prediction of the selected PDB code or docked complex and will communicate with the server to predict results in real-time. The “Clear” button resets the interface by clearing all the inputs and the displayed results, allowing users to start a new query. After any prediction, results will be shown at the bottom right of the interface inside the main display box. This box shows the predicted binding affinity, actual experimental binding affinity, and the ligand/protein files used for the prediction. For docked complexes, the display also includes the binding affinity predicted by AutoDock Vina 1.1.2 for comparison. All affinity values are presented in −log (Kd/Ki) (Figure 7). The model and all of the datasets are hosted on a separate server to guarantee an efficient user experience and save the user up to 8 GB of local storage space. The GUI is freely available for both Mac and Windows OS. The GNNSeq can be downloaded at https://github.com/sivaGU/GNNSeq.

## 3. Materials and Methods

### 3.1. Data Collection

Binding affinity data for training and testing was retrieved from the PDBbind v.2020 database [40]. We downloaded the “refined set”, which is made up of 5320 protein–ligand complexes with known binding affinities. The refined set contains binding affinity values expressed as either K_d_ dissociation constants or K_i_ inhibition constants. The “general set” is a more diverse set consisting of 14,127 protein–ligand complexes expressed in IC_50_, K_d_, and K_i_ units. Since our model only predicts the binding affinities using the negative base-10 log of the K_d_ and K_i_ values (pK_d_ and pK_i_), we removed the complexes in the “general set” that were present in only IC_50_ values. GNNSeq training and testing were performed using the “refined set” and the “general set” individually, as well as a “combined set” which is a shuffled merge of the “refined” and “general” sets. These datasets are widely used in drug discovery because they provide diverse and high-quality data [12]. The binding affinities serve as the target variable for the predictive model. Although four files were provided for each protein–ligand complex in each set from PDBbind, our model only used the .pdb file corresponding to the protein binding pocket and the .mol2 file for the ligand information. These files contain detailed atomic and connectivity information, which are necessary for accurate feature extraction [40]. The binding affinities were extracted and stored in a dictionary for efficient retrieval during the training and testing phases. We used the DUDE-Z v.2023.06.20 dataset for the external validation phase [22]. This dataset provides a large variety of active and decoy ligands in .mol2 format, as well as .pdb files for 43 receptors [22,41]. Building off of the original DUD-E dataset, DUDE-Z v.2023.06.20 introduces a 250,000-size collection of diverse decoy molecules that resemble active ligands [22].

### 3.2. Ligand and Protein Data Preprocessing

Once the data were collected, ligand structures from the complexes were converted to graphs using NetworkX v.3.4, a powerful library used for creating and manipulating complex graphs [42]. This conversion involves reading the atomic positions and bonds from the ligand PDB files and representing them as nodes and edges in a graph, respectively. Features such as node degrees, clustering coefficients, and betweenness centrality were extracted from these graph-like structures. The graph features provide a detailed representation of the ligand’s basic structural properties, which are necessary for accurate binding affinity predictions.

The protein pocket sequences were derived from residues in the pocket.pdb files, within 6 Å of the bound ligand, as identified using BioPython v.1.84’s structural parsing tools [43]. For each complex, the protein pocket structure was loaded with BioPython v.1.84’s PDB module, and residues within 6 Å of the ligand’s atomic coordinates were classified as part of the binding pocket. The resulting 3-letter pocket residue codes were converted to one-letter amino acid codes using BioPython v.1.84 and a custom one-hot encoding scheme. Protein pocket sequences were processed using BioPython v.1.84’s ProteinAnalysis function to derive a range of features, including amino acid frequencies, hydrophobicity, hydrophilicity, polarity, charge, molar extinction coefficient, and secondary structure fractions [43]. This processing involved converting the three-letter amino acid codes to one-letter codes and calculating various sequence-based features that describe the protein’s physicochemical properties. Protein features were further extracted using BioPython v.1.84, a powerful library for biological computation that provides tools for parsing biological file formats, analyzing sequences, and performing various biological tasks [43]. Sequence features allow us to capture the complex characteristics and pattern-based properties of proteins, which are fundamental for predicting binding affinity. After initial preprocessing, the protein sequences and ligand SMILES strings were one-hot encoded to create uniform feature representations. Padding was applied to ensure consistency in feature vector lengths. Applying one-hot encoding allows GNNSeq to process sequence data effectively. This step is important as it standardizes the input data and ensures that the model can handle sequences of varying lengths.

### 3.3. Feature Extraction

After preprocessing the proteins and ligands, chemical descriptors were extracted using RDKit v. 2024.03.4 [44]. The descriptors extracted include molecular weight, hydrogen bond donors and acceptors, LogP, and topological polar surface area. Additionally, a comprehensive set of ligand features was derived from RDKit v. 2024.03.4, covering various structural and chemical properties. These descriptors provide a quantitative view of the ligand’s chemical properties, which are important for identifying learnable patterns. Ligand features were extracted in three main categories: graph features, chemical descriptors, and additional ligand features (Table 10). The graph features include the number of nodes, number of edges, mean degree, clustering coefficient, and betweenness centrality. These features are essential for understanding the ligand’s structural properties and their impact on binding affinity. The chemical descriptors provide a quantitative measure of the ligand’s chemical properties, such as its mass, hydrophobicity, and the ability to form hydrogen bonds. The additional ligand features include descriptors provided by RDKit v. 2024.03.4 [44]. The number and type of ligand features were determined and optimized through extensive training and testing of GNNSeq. These features provide a detailed representation of the ligand’s structural and chemical properties, which are needed for the GNNSeq model to learn.

Similar to the ligands, protein features were extracted in two main categories: sequence features and additional protein features (Table 11). Sequence features, such as sequence length, amino acid frequencies, hydrophobicity, hydrophilicity, polarity, charge, molar extinction coefficient, isoelectric point, and secondary structure fraction, were extracted. These features provide a detailed representation of the protein’s physicochemical properties, which are necessary for learning patterns and sequences that influence ligand binding affinity. The additional protein features include aromaticity, instability index, flexibility, aliphatic index, grand average of hydropathy, molecular weight, charge composition, polar amino acid fraction, basic amino acid fraction, acidic amino acid fraction, turns fraction, beta-sheet fraction, alpha-helix fraction, disulfide bonds, and transmembrane helices. By understanding these properties, GNNSeq can make more accurate binding affinity predictions.

### 3.4. Dimensionality Reduction Using PCA and Outlier Removal

Once all the necessary features were extracted, Principal Component Analysis (PCA) was used to reduce the dimensionality of the feature space. This optimized computational efficiency and improved model performance. PCA transforms the original features into linearly uncorrelated components, which capture the maximum variance in the data with fewer dimensions [45]. This transformation reduces the risk of overfitting by eliminating redundant and noisy features. Reducing dimensionality accelerates the training process and increases the generalizability of GNNSeq. Applying PCA to our model was critical in managing the complexity of the feature sets derived from protein and ligand sequences. In an effort to optimize computational efficiency and improve model performance, we applied the scikit-learn library’s standard PCA function to the preprocessed features. For our analysis, we retained only the first two principal components (PC1 and PC2), as they collectively captured the majority of the variance in the data while significantly reducing dimensionality. This was carried out so that the model could efficiently process data without being overwhelmed by redundant information.

After PCA was applied, outliers were identified and removed using a combination of linear regression and Z-score filtering. First, a linear regression model was fitted to the experimental binding affinity values, establishing a trendline to represent the expected affinity distribution. Residuals, defined as the difference between the actual affinity values and their trendline-predicted counterparts, were then computed. To identify extreme deviations, a Z-score was calculated for these residuals, with the threshold set at a Z-score value outside ±3. Any complexes with residuals outside this range were removed from the dataset, ensuring that the training data remained representative of general trends while mitigating the risk of overfitting. We confirmed that the removed outliers were not concentrated in any specific protein or ligand class, preserving the diversity and generalizability of the dataset. To preserve the overall trends and relationships in the data, we monitored the Pearson correlation coefficient (PCC) before and after outlier removal. This approach differs from using the model’s predicted binding affinities for outlier detection. Instead, it ensures that only experimental affinities are considered in determining extreme deviations, making the dataset more reliable for training. In total, 29 complexes were removed from the 5320 protein–ligand complexes in PDBbind v.2020 based on this criterion.

These outliers were excluded to improve model performance and ensure that the data used for training were both accurate and reliable. Outliers can distort the results of predictive models by introducing bias and increasing variance. Typically, this leads to overfitting and poor generalization of unseen data. By removing outliers, GNNSeq focuses on patterns that are more representative of the general trends in the data. This improves the model’s reliability. The removal process was necessary to prevent extremes from skewing the model’s predictions. The approach allowed the model to better understand the relationship between protein–ligand complexes and their binding affinities. The goal was to create a stable and effective predictive model that could learn patterns from only significant data.

### 3.5. GNN Model Architecture

**Figure 8:**
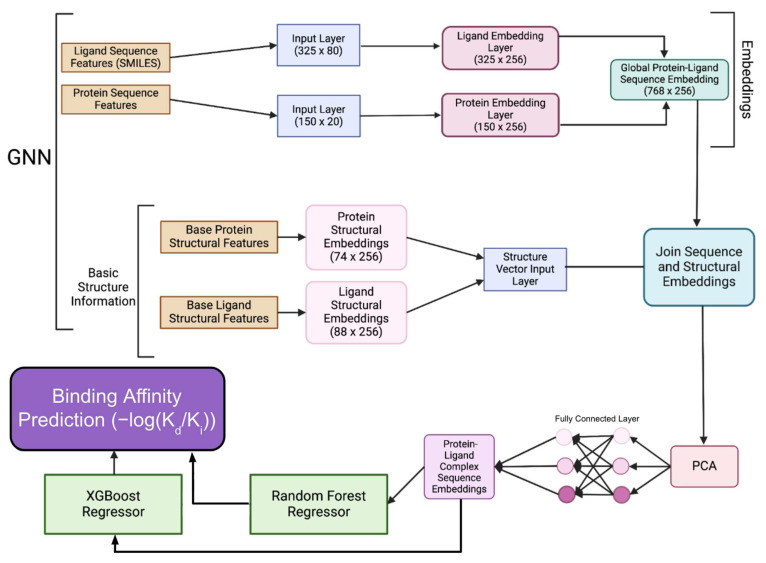
Architecture of GNNSeq. By processing ligand and protein sequence characteristics through embedding layers and combining them with structural features, GNNSeq uses a hybridized GNN architecture to predict binding affinities. The GNNSeq architecture starts by mapping the features of the protein and ligand sequences (SMILES) to specific dimensional representations by encoding them through distinct input layers. The high-level ligand (325 × 256) and protein (150 × 256) embeddings are created by passing these features through embedding layers. After that, structural information is processed independently to provide structural embeddings for proteins (74 × 256) and ligands (88 × 256). An integrated representation is created by combining the sequence and structural embeddings through a structure vector input layer. The integrated embeddings are refined into protein–ligand complex embeddings through a completely connected layer. To reduce dimensionality and improve efficiency, PCA is applied, condensing critical features from sequence and structural data. The condensed representation is passed to XGBoost and Random Forest regressors, which independently predict binding affinities. The final predictions are made by averaging the outputs of two ensemble models (XGBoost and Random Forest) that separately predict binding affinities using the fine-tuned embeddings. This hybrid approach optimizes predictive accuracy and efficiency by combining GNN-based embeddings with ensemble learning. This architecture is further explained in Section 3.5.

GNNSeq uses a hybridized GNN architecture to preprocess and predict binding affinities (Figure 8). The model begins by encoding the ligand sequence features (SMILES) and protein sequence features through separate input layers, using the PyTorch Python library v.2.5.1 to create the layers. The ligand input layer maps a (325 × 80) dimensional representation and the protein input layer maps a (150 × 20) dimensional representation. These encoded sequences are passed through dedicated embedding layers, ultimately producing the ligand embedding layer (325 × 256) and protein embedding layer (150 × 256) that capture high-level features relevant to each sequence. Once the embedding layers are created, basic structural information for both protein and ligand is processed separately to generate protein structural embeddings (74 × 256) and ligand structural embeddings (88 × 256). These structural features are vectorized and combined via the structure vector input layer, which joins both the sequence-based and structural embeddings into an integrated representation. The resulting embeddings are then processed through the global protein–ligand sequence embedding layer that condenses the information into a comprehensive feature vector.

When all these embeddings are created, a fully connected layer refines these embeddings to create the protein–ligand complex sequence embeddings. To reduce the dimensionality and improve model efficiency, PCA is applied to create a vector that encapsulates the most important features from both sequence and structural information. This condensed representation is then passed to a regressor consisting of XGBoost and Random Forest, which predicts the binding affinity. The features extracted by the GNN from the protein–ligand sequence and structural embeddings are concatenated with other chemical descriptors and sequence features, then padded and filtered for uniformity. The padded feature set is passed as input to both the RF and XGBoost models. The individual outputs from RF and XGBoost are averaged to achieve the final binding affinity prediction. This hybridized structure combines GNN-based sequence embeddings with ensemble learning to optimize accuracy and efficiency.

After developing the base model of GNNSeq, we created a collection of hybrid models that use GNNSeq to increase the binding affinity prediction accuracy. These hybrid models differ from GNNSeq mostly in the features they give priority to. GNNSeq focuses on sequence-based features, while the other two models are designed to capture structure and interaction-based features. The interaction-based model uses a GNN to extract features from the interactions between the SMILES representation of the ligand and the binding pocket of the protein. On the other hand, the structural model generates separate graphs for the ligand and protein, combines their structural features, and processes them using a GNN.

### 3.6. Hyperparameter Optimization with Hyperopt

To further improve the predictive performance of the model, hyperparameter optimization was conducted using Hyperopt v.0.2.7 [38]. Hyperopt is a powerful optimization framework that searches for the best hyperparameters within a broad range by using probabilistic models. We used Hyperopt v.0.2.7 because of its ability to procedurally explore the hyperparameter space and identify optimal parameters that maximize the model’s accuracy and efficiency. The search space for hyperparameters was defined separately for the GNN, the Random Forest Regressor, and the XGBoost model. In terms of the Random Forest Regressor, the number of estimators (rf_n_estimators) was allowed to vary between 50 and 500, and the maximum depth (rf_max_depth) was between 5 and 50. For the XGBoost model, the number of estimators (xgb_n_estimators) varied between 50 and 500, the maximum depth (xgb_max_depth) between 3 and 15, the learning rate (xgb_learning_rate) between 0.01 and 0.2 on a logarithmic scale, and the subsample ratio (xgb_subsample) between 0.6 and 1.0.

Hyperopt’s objective function was designed to train all 3 sub-models with the given hyperparameters and evaluate their performance using ten-fold cross-validation [38]. The primary metric for optimization was the Pearson Correlation Coefficient (PCC). A higher PCC indicates a stronger correlation, which indicates a better predictive performance. The average PCC across the folds was used to guide the optimization process, with the goal of maximizing the PCC. Hyperopt v.0.2.7 was configured to use the Tree-structured Parzen Estimator (TPE) algorithm for suggesting hyperparameter values. The optimization process was set to run for a maximum of 10 evaluations [38]. During the optimization, the PCC without Hyperopt was 0.77. At 50% completion, the best PCC achieved was approximately 0.787, which showed an improvement of 0.01. This suggests that while Hyperopt improved model performance, the improvement was relatively negligible. Once optimization was completed, the best hyperparameters identified by Hyperopt were used to define the final GNN, Random Forest, and XGBoost models. These models were trained and evaluated using ten-fold cross-validation.

### 3.7. Hybridizing GNNSeq with a CNN Framework

Once the model was optimized, we integrated Convolutional Neural Networks (CNNs) into the hybridized GNNSeq to use the spatial hierarchies and local patterns inherent in both protein and ligand structures [6,9,46]. CNNs are generally effective in understanding spatial dependencies and structural ideas, which become important when trying to understand complex patterns between proteins and ligands. By combining the strengths of GNNSeq with a CNN, the hybrid model can achieve a more holistic view of the learning needed to improve predictions. The CNN framework is designed to process both protein and ligand structural information to analyze their spatial relationships and hierarchical features. The architecture consists of several layers, each performing specific functions to extract and process features from the input data. The input layer accepts three-dimensional structural data of the protein–ligand complex, represented as a 3D grid where each point contains information about the presence of specific atoms and their types. Convolutional layers apply a series of convolution operations to the input data using learnable filters to detect local patterns and features. ReLU (Rectified Linear Unit) activation functions are applied after each convolutional layer to introduce non-linearity, which allows the CNN to capture more complex relationships between the input features. Pooling layers, particularly max pooling, are used to simplify the input by selecting the maximum value from each sub-region of the input grid. This reduces computational load and the number of parameters while retaining important features. After a series of convolutional and pooling layers, the output is flattened and fed into fully connected layers that analyze the features extracted from earlier layers. These layers consolidate the learned features and provide the final binding affinity prediction.

The CNN framework is trained using the same 3 datasets of protein–ligand complexes as GNNSeq, the “refined”, “general”, and “combined” sets. The training process uses backpropagation and gradient descent to minimize the loss function, which measures the differences between the predicted and actual binding affinities. The mean squared error (MSE) loss function is used to show the difference between the predicted and actual binding affinities. The Adam optimizer is used to adjust the model parameters during training. The CNN was developed to process the atomic structural information and serve as another layer in processing the embeddings from the GNN model. The CNN framework was integrated into GNNSeq to create a unified architecture that combines the strengths of both approaches. The embeddings generated by the GNN were passed to the CNN framework, where they were processed through convolutional layers to extract spatial and hierarchical features. They were then merged into a unified architecture that uses the strengths of both models. The extra features extracted by the CNN from the 3D structural data are concatenated with the sequence and graph-based features. The entire model is optimized using Hyperopt v.0.2.7 to provide the best possible predictions. The performance of the integrated hybrid model is evaluated using PCC, MSE, and MAE.

## 4. Limitations

GNNSeq performed well in predicting protein–ligand binding affinities but has some limitations that must be addressed to potentially increase the model’s accuracy and robustness. Although it is the purpose of this study, the most significant weakness is the model’s sole reliance on sequence-based features alone in developing the predictors, without consideration of any direct spatial interactions between proteins and ligands. These spatial interactions are very helpful in predicting binding affinity, especially when the 3D conformation and orientation of the molecules play a key role. Protein–ligand interactions, such as hydrogen bonds, van der Waals forces, and electrostatic interactions, are crucial in determining binding affinity. These are important when it comes to complex binding scenarios where spatial orientation and specific interaction sites are key determinants. Since interaction features are excluded, this may lead to the model suffering from incomplete understanding of the binding mechanisms. This could lower the accuracy of the model’s predictions if given very diverse and unseen data. GNNSeq was designed as a sequence-based model to ensure adaptability to various input formats and data qualities. By focusing plainly on sequence-based features and patterns, it eliminates the need for pre-docked complexes as input. The program can process raw .pdb and .pdbqt files through comprehensive sanitization and preprocessing. Although this approach sacrifices interaction-specific details, it enables GNNSeq to remain highly flexible and deliver competitive predictions across diverse datasets.

Another significant limitation is the process of outlier removal. While necessary for enhancing GNNSeq’s learning ability, it can introduce biases into the dataset. Outliers are removed using a Z-score threshold of ±3 to eliminate extreme values that could skew model predictions. However, this approach may exclude data points with substantial variances, potentially reducing the model’s capacity to generalize. Additionally, integrating CNNs into the proposed model presents its own set of challenges. While CNNs are good for processing spatial information, their inclusion has reduced the efficiency of GNNSeq. The CNN-enhanced hybrid models ran three to four times slower than those that did not use CNNs, which is a considerable limitation when dealing with much larger datasets. In addition, the accuracy of the CNN-integrated models was worse compared to other hybrid combinations. This shows that even though CNNs are helpful in capturing complex spatial patterns, the additional information did not translate into improved predictions.

## 5. Future Directions

GNNSeq architecture was developed to identify intricate sequence-based patterns in protein–ligand complexes that determine binding affinity. Future work will focus on several areas to overcome GNNSeq’s limitations and improve its prediction abilities. Optimizing the merging strategies with structure and interaction-based models could significantly improve accuracy without risking overfitting. Higher prediction accuracy may be possible with further improvement in this area, especially considering the trend of rising PCC observed as we refined our merging strategies. These hybridized models show great potential for advancing de novo predictions and virtual screening, particularly in scenarios where precise 3D conformational data are unavailable. Building on these findings, future development will focus on integrating docking methods into the GNNSeq + Int_alg_ + Struct_alg_ framework to improve predictions of any protein–ligand complex that enable it to perform large-scale virtual drug discovery screening. Additionally, we will prioritize testing the optimized merged models on a broader range of de novo ligands with known experimental binding affinities to validate their ability to generalize to novel compounds.

Aside from addressing the hybridized models, optimizing the CNN architecture could potentially increase prediction accuracy. While the inclusion of CNNs slowed down the current GNNSeq, refining the CNN layers by using advanced techniques such as attention mechanisms or residual connections could improve the model’s ability to capture spatial hierarchies without compromising efficiency. Fine-tuning the CNN’s hyperparameters might address issues of overfitting as well. These changes have the potential to improve the model’s ability to handle de novo compounds without prior binding data. However, the inclusion of more diverse de novo ligands, such as PFAS, presents a significant opportunity to improve GNNSeq’s prediction capabilities. Finally, the GNNSeq GUI does not allow users to input their own PDB complexes, such as ligand and protein files, for testing. Direct file uploads can be challenging due to file size limitations and the need for secure processing to prevent issues such as server downtime caused by incorrectly formatted files. In future work, we aim to include this functionality by adding server-side capabilities to support user-uploaded data.

Another important consideration for future development is how GNNSeq compares to more computationally expensive biophysics-based methods, such as all-atom molecular dynamics (MD) simulations, in terms of accuracy and efficiency. All-atom MD simulations are among the most computationally intensive and quantitatively accurate methods for predicting ligand–receptor binding affinity. Their ability to model atomic interactions, solvent effects, and thermodynamic fluctuations in a dynamic environment makes them highly detailed and accurate. However, the significant computational cost and long simulation times required for adequate sampling limit their practical use, particularly in large-scale drug discovery applications. GNNSeq provides a computationally efficient alternative by using sequence-based features and a hybrid GNN framework integrated with Random Forest and XGBoost models. Unlike MD, which explicitly samples protein–ligand conformational dynamics, GNNSeq predicts binding affinity by extracting molecular patterns from experimental data. This allows for rapid predictions while maintaining competitive accuracy. Benchmarking results show that GNNSeq achieves high predictive performance (PCC = 0.784 on the PDBbind refined set and 0.84 on the core set), and external validation using the DUDE-Z v.2023.06.20 dataset demonstrated its ability to distinguish active ligands from decoys (AUC = 0.74). GNNSeq model efficiency and robustness make it a valuable tool for large-scale screening. One limitation of GNNSeq is its reliance on static sequence-derived features, which do not explicitly account for ligand–receptor flexibility. In contrast, MD simulations provide detailed insights into protein–ligand interactions by considering multiple conformational states, albeit at a much higher computational cost. While GNNSeq is significantly faster than MD, incorporating conformational flexibility into the model could further improve its predictive accuracy.

Thus, our future studies will be on potential improvements to GNNSeq involving incorporating these protein–ligand dynamics by sampling multiple conformers of protein–ligand complexes obtained from short molecular dynamics (MD) simulations. Instead of relying on a single static structure, training the model with conformers that capture protein and ligand flexibility may increase its ability to generalize across diverse binding environments. In this approach, short MD simulations (e.g., 1–10 ns) would be performed using MD simulation software. Snapshots of the protein–ligand complexes would be extracted at regular intervals (e.g., every 10–100 ps) to capture conformational variations. These multiple conformers would then be integrated into GNNSeq’s training framework, which allows the model to learn from dynamic structural variations rather than a single static representation. The expanded dataset, incorporating multiple conformers, would enable GNNSeq to recognize binding affinity patterns that arise from molecular flexibility. The predictive performance of the model would be evaluated by comparing results before and after incorporating conformer-based training, using various statistical metrics. If this approach proves effective, it could lead to a hybrid framework that retains the computational efficiency of GNNSeq while improving its predictive power by incorporating key aspects of structural flexibility observed in MD simulations. This strategic approach represents a logical step toward bridging the gap between fast, sequence-based machine learning models and physics-based simulations and improving GNNSeq’s applicability in real-world drug discovery scenarios.

## 6. Conclusions

In this study, we developed and externally validated GNNSeq, a novel hybrid model that combines a Graph Neural Network (GNN) with Random Forest and XGBoost to predict protein–ligand binding affinity based solely on sequence features. GNNSeq is a robust solution for binding affinity prediction when sufficient structural and protein–ligand interaction data are unavailable. The sequence protein and ligand features were extracted using RDKit v. 2024.03.4 and BioPython v.1.84. PCA was used to further reduce the high dimensionality of these features. GNNSeq was trained and tested on subsets of the PDBbind dataset, achieving a PCC of 0.784 on the PDBbind v.2020 refined set, which displayed its ability to accurately predict binding affinity without relying on structural or interaction data. GNNSeq competes with other related machine learning models that use many more complex extracted features. External validation on the DUDE-Z v.2023.06.20 dataset demonstrated GNNSeq’s robustness, as it successfully distinguished between active ligands and decoys across diverse ligand–receptor pairs, with an average AUC of 0.74. These results show GNNSeq’s versatility and potential for applications where structural information is limited. This is supported by its efficient runtime and scalable design. Overall, GNNSeq provides an effective and reliable approach for binding affinity prediction, with demonstrated improvements in accuracy and generalizability over existing sequence-based models. The model’s ability to generalize to diverse datasets makes it highly suitable for predicting binding affinities of de novo complexes in future studies.

Beyond its immediate predictive capabilities, GNNSeq has broader implications for drug discovery, particularly in the early stages where interaction and structural data are often unavailable. By predicting binding affinities based solely on sequence features, GNNSeq can accelerate the identification of novel drug candidates by screening millions or even billions of compounds, reducing reliance on costly and time-consuming structural characterization methods. With its high computational efficiency, GNNSeq can rapidly process large datasets, further mitigating the time constraints typically associated with early-stage drug discovery. This makes it a valuable tool for large-scale virtual screening and hit identification, providing an efficient and cost-effective approach to drug development. Future work will focus on fine-tuning GNNSeq through improved merging strategies with structure- and interaction-based models. Preliminary results indicate that optimized merging techniques have the potential to further increase prediction accuracy without introducing overfitting. These future steps will aim to expand GNNSeq’s ability to predict binding affinities across diverse datasets.

## Figures and Tables

**Table 1:** Averaged 10-fold cross-validation results on the refined set.

R^2^ Score	MSE (kcal/mol)	MAE (kcal/mol)	PCC	AUC
0.595	1.524	0.963	0.784	0.792

**Table 2:** Averaged 10-fold cross-validation results on the general set.

R^2^ Score	MSE (kcal/mol)	MAE (kcal/mol)	PCC	AUC
0.479	1.885	1.068	0.718	0.760

**Table 3:** Averaged 10-fold cross-validation results on the combined set.

R^2^ Score	MSE (kcal/mol)	MAE (kcal/mol)	PCC	AUC
0.5091	1.778	1.039	0.7401	0.7765

**Table 4:** External validation of GNNSeq using the refined and general sets.

Model	PCC	MSE (kcal/mol)	MAE (kcal/mol)	R^2^	AUC
GNNSeq_1_	0.612	2.512	1.273	0.373	0.721
GNNSeq_2_	0.687	2.137	1.091	0.461	0.743

**Table 5:** Benchmarking of GNNSeq against deep learning methods on PDBbind datasets.

Machine Learning Method	Test Dataset of Protein–Ligand Complexes	PCC	MSE (kcal/mol)	MAE (kcal/mol)
GNNSeq	PDBbind v.2020 Refined Set	0.784	1.51	0.957
GNNSeq	PDBbind v.2019 Refined Set	0.771	1.56	0.988
GNNSeq	PDBbind v.2016 Refined Set	0.768	1.58	1.048
GNNSeq	PDBbind v.2016 Core Set	0.839	1.665	1.001
GNNSeq	PDBbind v.2013 Core Set	0.749	1.63	1.062
HNN_affinity_ (Sequence + SMILES) [9]	PDBbind v.2019 Refined Set	0.830	1.04	-
Random Forest-Based [21]	PDBbind v.2019 Refined Set	0.779	1.56	-
Random Forest-Based [23]	PDBbind v.2016 Refined Set	0.776	1.58	-
X-Score::HMScore [23]	PDBbind v.2016 Refined Set	0.644	-	-
SS-GNN Refined Set [24]	PDBbind v.2013 Core Set	0.795	-	1.16
Pafnucy [24]	PDBbind v.2016 Core Set	0.780	1.420	-
SIGN [24]	PDBbind v.2016 Refined	0.797	1.316	-
OnionNet [24]	PDBbind v.2013 Core Set	0.782	1.503	-
MP-GNN [24]	PDBbind v.2013 Core Set	0.805	0.801	-
PerSpect-ML [24]	PDBbind v.2013 Core Set	0.793	1.956	-
SS-GNN General Set [24]	PDBbind v.2013 Core Set	0.815	1.347	1.16
HAC-Net [25]	PDBbind v.2016 Core Set	0.846	-	0.971
TopBP [26]	PDBbind v.2016 Core Set	0.861	-	-
AEScore [27]	PDBbind v.2016 Core Set	0.83	-	-
AK-score [28]	PDBbind v.2016 Core Set	0.812	-	-
BAPA [29]	PDBbind v.2016 Core Set	0.819	-	1.021
GraphBAR [30]	PDBbind v.2016 Core Set	0.726	-	1.241
CAPLA [16]	PDBbind v.2016 Core Set	0.843	-	0.966
EHIGN [31]	PDBbind v.2016 Core Set	0.854	-	-
T-ALPHA [32]	PDBbind v.2016 Core Set	0.869	-	0.875
TopoFormer-Seq [33]	PDBbind v.2016 Core Set	0.864	-	-
MFE [34]	PDBbind v.2016 Core Set	0.851	-	0.882
LGN [35]	PDBbind v.2016 Core Set	0.842	-	0.936
GIGN [36]	PDBbind v.2016 Core Set	0.840	-	-
Hydrascreen [37]	PDBbind v.2016 Core Set	0.86	-	-

**Table 6:** Comparative performance of GNNSeq algorithmic hybrids.

Unique Combination of Refined Set (Algorithmic Hybrid)	PCC	AUC	Time Elapsed Using Best Parameters (Without Hyperopt)
CNN + GNN	0.72	0.74	6 h 6 min
CNN	0.66	0.72	3 h 37 min
GNN + XGBoost + RF	0.784	0.792	1 h 32 min
GNN + CNN + XGBoost	0.744	0.76	6 h 47 min
GNN + CNN + RF	0.741	0.73	6 h 41 min
CNN + XGBoost + RF	0.687	0.70	6 h 8 min
GNN + CNN + XGBoost + RF	0.783	0.749	8 h 49 min

**Table 7:** Metrics from merging strategies of GNNSeq with an interaction-based model.

Merge Version	PCC	AUC
GNNSeq—Standalone Model	0.784	0.792
GNNSeq + *Int_alg_* (V1): Embedding Concatenation	0.788	0.780
GNNSeq + *Int_alg_* (V2): Stacking (4 layers)	0.792	0.790
GNNSeq + *Int_alg_* (V3): Kernel-level optimizer	0.802	0.790
GNNSeq + *Int_alg_* (V4): Kernel-Level Optimizer on Stacking (4 layers)	0.826	0.805

**Table 8:** Metrics from merging strategies of GNNSeq with a structure-based model.

Merge Version	PCC	AUC
GNNSeq—Standalone Model	0.784	0.792
GNNSeq + *Struct_alg_* (V1): Embedding Concatenation	0.721	0.741
GNNSeq + *Struct_alg_* (V2): Stacking (4 layers)	0.748	0.770
GNNSeq + *Struct_alg_* (V3): Kernel-level optimizer	0.759	0.790
GNNSeq + *Struct_alg_* (V4): Kernel-Level Optimizer on Stacking (4 layers)	0.794	0.802

**Table 9:** Outcomes of merging strategies of GNNSeq with interaction and structure-based models.

Merge Version	PCC	AUC
GNNSeq: Standalone Model	0.784	0.792
GNNSeq + *Int_alg_* + *Struct_alg_* (V1): Embedding Concatenation	0.789	0.772
GNNSeq + *Int_alg_* + *Struct_alg_* (V2): Stacking (7 layers +1 hidden layer)	0.821	0.804
GNNSeq + *Int_alg_* + *Struct_alg_* (V3): Kernel-level optimizer	0.828	0.815
GNNSeq + *Int_alg_* + *Struct_alg_* (V4): Kernel-Level Optimizer on Stacking (8 layers)	0.839	0.819

**Table 10:** List of sequence-based ligand features used in the GNNSeq model.

Feature Name	Description
**Graph Features**	
Number of Nodes	Total number of atoms in the ligand molecule.
Number of Edges	Total number of bonds in the ligand molecule.
Mean Degree	Average number of bonds per atom.
Clustering Coefficient	Tendency of atoms to form clusters.
Betweenness Centrality	Importance of atoms in maintaining shortest paths.
**Chemical Descriptors**	
Molecular Weight	Total mass of the molecule.
Number of Hydrogen Bond Donors	Atoms that can donate hydrogen bonds.
Number of Hydrogen Bond Acceptors	Atoms that can accept hydrogen bonds.
LogP	Hydrophobicity of the molecule.
Topological Polar Surface Area (TPSA)	Surface area of polar atoms.
**Additional Ligand Features**	
NumAliphaticCarbocycles	Count of aliphatic carbocycles.
NumAliphaticHeterocycles	Count of aliphatic heterocycles.
NumAliphaticRings	Count of aliphatic rings.
NumAromaticCarbocycles	Count of aromatic carbocycles.
NumAromaticHeterocycles	Count of aromatic heterocycles.
NumAromaticRings	Count of aromatic rings.
FractionCSP3	Fraction of sp3 hybridized carbons.
HeavyAtomCount	Number of non-hydrogen atoms.
NHOHCount	Number of nitrogen, hydrogen, oxygen, and hydroxyl groups.
NOCount	Number of nitrogen and oxygen atoms.
NumHeteroatoms	Number of non-carbon atoms.
NumRadicalElectrons	Number of radical electrons.
NumSaturatedCarbocycles	Count of saturated carbocycles.
NumSaturatedHeterocycles	Count of saturated heterocycles.
NumSaturatedRings	Count of saturated rings.
RingCount	Total number of rings.
MolMR	Molar refractivity.
qed	Quantitative estimate of drug-likeness.
PEOE_VSA1 to PEOE_VSA14	Partial charge surface area descriptors.
SMR_VSA1 to SMR_VSA8	Molar refractivity surface area descriptors.
SlogP_VSA1 to SlogP_VSA12	LogP surface area descriptors.
VSA_EState1 to VSA_EState10	Electrostatic state surface area descriptors.

**Table 11:** List of sequence-based protein features used in the GNNSeq model.

Feature Name	Description
**Sequence Features**	
Sequence Length	Total number of amino acids.
Amino Acid Frequencies	Proportion of each amino acid type.
Hydrophobicity	Average hydrophobicity.
Hydrophilicity	Average hydrophilicity.
Polarity	Average polarity.
Charge	Average charge.
Molar Extinction Coefficient	Measure of how strongly the protein absorbs light at a particular wavelength.
Isoelectric Point	pH at which the protein has no net charge.
Secondary Structure Fraction	Fraction of alpha-helix, beta-sheet, and random coil structures.
**Additional Protein Features**	
Aromaticity	Relative frequency of aromatic amino acids.
Instability Index	Stability of the protein.
Flexibility	Average flexibility.
Aliphatic Index	Volume occupied by aliphatic side chains.
Gravy (Grand Average of Hydropathy)	Average hydropathy value.
Isoelectric Point (pI)	pH at which the protein has no net charge.
Molecular Weight	Total mass of the protein.
Charge Composition	Ratio of positively charged to negatively charged amino acids.
Polar Amino Acid Fraction	Fraction of polar amino acids.
Basic Amino Acid Fraction	Fraction of basic amino acids.
Acidic Amino Acid Fraction	Fraction of acidic amino acids.
Turns Fraction	Fraction of amino acids in turns.
Beta-Sheet Fraction	Fraction of amino acids in beta-sheets.
Alpha-Helix Fraction	Fraction of amino acids in alpha-helices.
Disulfide Bonds	Number of disulfide bonds.
Transmembrane Helices	Prediction of transmembrane helices.

## Data Availability

GNNSeq was implemented in Python and is accessible for download and testing through the following link: https://github.com/sivaGU/GNNSeq. The repository contains all necessary files for running the GNNSeq GUI model, along with detailed instructions for installation, usage, and interpretation of the model and its outputs. Please allow up to 3 min for the GUI to initially boot up and connect to the server. The above GitHub repository includes separate zip files containing the executables for the locally downloadable GNNSeq GUI for both Windows and macOS. It also includes a readme file that provides clear instructions for downloading and running the application.

## Supplementary Materials

The following supporting information can be downloaded at: https://www.mdpi.com/article/10.3390/ph18030329/s1. The Supporting Information is available free of charge. The Supplementary Materials folder contains the tables and XLSX files listed below. Table S1: Results of an example run of GNNSeq trained and tested on the refined set (XLSX); Table S2: Results per fold of 10-fold cross-validation on the refined set; Table S3: Results per fold of 10-fold cross-validation on the general set; Table S4: Results per fold of 10-fold cross-validation on the combined set; Table S5: Results of an example run of GNNSeq trained on the refined set and externally validated on the general set; Table S6: All the resultant values and individual violin plots for the DUDE-Z predictions across all 43 receptors (XLSX); Table S7: Predicted binding affinities from all hybridized GNNSeq models for the 12 protein–ligand complexes virtually docked by AutoDock Vina 1.1.2.
