# Supplementary material for: GNNSeq: A Sequence-Based Graph Neural Network for Predicting Protein–Ligand Binding Affinity
Source: Pharmaceuticals (Basel). 2025 Feb 26;18(3):329. doi: 10.3390/ph18030329 (PMC11945123; doi:10.3390/ph18030329)
Supplement: Supplementary file 1 [file pharmaceuticals-18-00329-s001.zip › Table S2.pdf]

Table S2. Results per Fold of 10-Fold Cross-Validation on the Refined Set.

Fold	R^2 Score	MSE (kcal/mol)	MAE (kcal/mol)	PCC	AUC
1	0.6129	1.418	0.944	0.7843	0.7691
2	0.5827	1.591	0.977	0.7649	0.7844
3	0.5914	1.522	0.980	0.7695	0.7844
4	0.5551	1.559	0.986	0.7455	0.7786
5	0.6650	1.342	0.888	0.8193	0.8260
6	0.5572	1.757	1.053	0.7492	0.7610
7	0.5866	1.496	0.951	0.7663	0.7859
8	0.6209	1.598	0.954	0.7898	0.8317
9	0.5846	1.489	0.939	0.7654	0.8050
10	0.5934	1.469	0.955	0.7708	0.7916
