# Supplementary material for: GNNSeq: A Sequence-Based Graph Neural Network for Predicting Protein–Ligand Binding Affinity
Source: Pharmaceuticals (Basel). 2025 Feb 26;18(3):329. doi: 10.3390/ph18030329 (PMC11945123; doi:10.3390/ph18030329)
Supplement: Supplementary file 1 [file pharmaceuticals-18-00329-s001.zip › Table S3.pdf]

Table S3. Results per Fold of 10-Fold Cross-Validation on the General Set.

Fold	R^2 Score	MSE (kcal/mol)	MAE (kcal/mol)	PCC	AUC
1	0.478	1.913	1.064	0.714	0.753
2	0.464	1.842	1.084	0.733	0.748
3	0.487	1.856	1.074	0.741	0.783
4	0.462	1.828	1.057	0.707	0.771
5	0.461	1.937	1.062	0.709	0.749
6	0.479	1.922	1.069	0.715	0.758
7	0.498	1.901	1.081	0.720	0.752
8	0.490	1.879	1.057	0.73	0.744
9	0.459	1.861	1.073	0.705	0.781
10	0.502	1.915	1.065	0.712	0.760
