# Supplementary material for: GNNSeq: A Sequence-Based Graph Neural Network for Predicting Protein–Ligand Binding Affinity
Source: Pharmaceuticals (Basel). 2025 Feb 26;18(3):329. doi: 10.3390/ph18030329 (PMC11945123; doi:10.3390/ph18030329)
Supplement: Supplementary file 1 [file pharmaceuticals-18-00329-s001.zip › Table S4.pdf]

Table S4. Results per Fold of 10-Fold Cross-Validation on the Combined Set.

Fold	R^2 Score	MSE (kcal/mol)	MAE (kcal/mol)	PCC	AUC
1	0.5088	1.783	1.034	0.7431	0.7834
2	0.5194	1.772	1.025	0.7467	0.7846
3	0.4947	1.784	1.063	0.7434	0.7639
4	0.5293	1.795	1.014	0.7398	0.7984
5	0.5284	1.728	1.083	0.7257	0.7649
6	0.5013	1.791	1.045	0.7535	0.7738
7	0.5076	1.772	1.037	0.7255	0.7761
8	0.4957	1.769	1.022	0.7344	0.7623
9	0.4830	1.802	1.035	0.7465	0.7845
10	0.5235	1.785	1.041	0.7431	0.7731
