# Supplementary material for: GNNSeq: A Sequence-Based Graph Neural Network for Predicting Protein–Ligand Binding Affinity
Source: Pharmaceuticals (Basel). 2025 Feb 26;18(3):329. doi: 10.3390/ph18030329 (PMC11945123; doi:10.3390/ph18030329)
Supplement: Supplementary file 1 [file pharmaceuticals-18-00329-s001.zip › Table S7.pdf]

Table S7. Table Showing the Exact Predicted Values that were Used for the Column Chart in Figure 7. All Values are Shown in -log(K_d/K_i)

PDBCode	GNNSeq	GNNSeq + Interaction	GNNSeq + Structure	GNNSeq + Interaction + Structure	Experimental Values
2kaw	5.29	4.95	5.19	5.06	4.97
7odn	5.78	6.12	5.48	5.44	5.14
3rx3	7.1	6.73	6.72	6.71	6.53
4qmz	6.66	6.5	6.73	6.52	6.47
4agd	8.2	8.15	8.34	8.23	8.41
6nfz	7.58	7.66	7.75	7.67	7.8
6jok	8.43	8.23	8.64	8.74	9.1
4ks8	6.79	6.84	6.71	7.1	5.62
4wev	7.03	6.37	6.15	6.05	5.57
3g0f	7.31	7.16	7.52	7.38	7.66
6ng0	7.5	7.34	8.06	7.74	8.19
3u2c	6.96	6.59	6.79	6.67	6.53
